# Incomplete contingency tables with censored cells with application to estimating the number of people who inject drugs in Scotland

**DOI:** 10.1002/sim.6047

**Published:** 2013-12-01

**Authors:** Antony M Overstall, Ruth King, Sheila M Bird, Sharon J Hutchinson, Gordon Hay

**Affiliations:** a School of Mathematics and Statistics, University of St AndrewsSt Andrews, U.K.; b Medical Research Council Biostatistics UnitCambridge, U.K.; c Department of Mathematics and Statistics, University of StrathclydeGlasgow, U.K.; d School of Health and Life Sciences, Glasgow Caledonian UniversityGlasgow, U.K.; e Health Protection ScotlandGlasgow, U.K.; f Centre for Public Health, Liverpool John Moores UniversityLiverpool, U.K.

**Keywords:** censoring, incomplete contingency table, people who inject drugs, log-linear models, population size

## Abstract

Estimating the size of hidden or difficult to reach populations is often of interest for economic, sociological or public health reasons. In order to estimate such populations, administrative data lists are often collated to form multi-list cross-counts and displayed in the form of an incomplete contingency table. Log-linear models are typically fitted to such data to obtain an estimate of the total population size by estimating the number of individuals not observed by any of the data-sources. This approach has been taken to estimate the current number of people who inject drugs (PWID) in Scotland, with the Hepatitis C virus diagnosis database used as one of the data-sources to identify PWID. However, the Hepatitis C virus diagnosis data-source does not distinguish between current and former PWID, which, if ignored, will lead to overestimation of the total population size of current PWID. We extend the standard model-fitting approach to allow for a data-source, which contains a mixture of target and non-target individuals (i.e. in this case, current and former PWID). We apply the proposed approach to data for PWID in Scotland in 2003, 2006 and 2009 and compare with the results from standard log-linear models. © 2013 The Authors. *Statistics in Medicine* published by John Wiley & Sons, Ltd.

## 1. Introduction

Estimating population sizes using two (or more) data lists has a long history 1. A number of underlying assumptions is typically made, including, for example, that individuals are uniquely identifiable by each data-source, so that individuals can be collated between the different data-sources. The corresponding data are most often displayed in the form of a contingency table, where each cell corresponds to the number of individuals observed by each combination of data-sources. However, the table is ‘incomplete’ in that the number of individuals not observed by any of the data-sources is unknown. Here, we distinguish our notion of incomplete contingency tables from those that contain structural zeros 2 and those that are partially classified 3.

Traditionally, log-linear models 4 have been fitted to the observed cells, modelling the probability of being observed by each cross-classification of data-sources and permitting the estimation of the unknown cell count, which can be combined with the total number of observed individuals to provide an estimate of the total population size. Log-linear models are composed of main-effect terms and interactions terms between the different data-sources. Typically, the underlying log-linear model is unknown, and competing models (in terms of the interactions present in the models) can lead to very different estimates of the total population size. Model discrimination is, therefore, an important component of the statistical analysis. Madigan and York 5 and King and Brooks 6 proposed Bayesian model-averaging approaches, incorporating both parameter and model uncertainty within the estimation of the total population size.

Log-linear models have been extended to allow additional (discrete) covariates to be incorporated 7–12. Specifically, the incomplete contingency table can be extended so that individuals are not only cross-classified according to the combination of data-sources they are observed by but also according to the different covariate levels. In such tables, there are multiple unknown cell counts, corresponding to the number of individuals not observed by any of the data-sources for each cross-classification of possible covariates. Log-linear models fitted to such data are accordingly extended, allowing for additional main-effects for each covariate plus interactions between covariates and/or data-sources.

The fact that a cell count of the table is unknown, or missing, may motivate us to consider the missing data mechanism 13. This is where the probability of a response being observed (or unobserved) is considered, including its relationship to the parameters and observed responses. This leads to the terms missing completely at random, missing at random and missing not at random. Proper consideration of these mechanisms is important for valid inference. However, in our concept of an incomplete contingency table, the probability of a response being observed (or unobserved) by each combination of data-sources is actually what we are modelling with log-linear models. For example, an interaction between two data-sources means that the probability of being observed by one of the data-sources either increases or decreases depending on whether the other data-source observed the individual.

In this paper, we relax the standard assumption that all individuals recorded by the given data-sources belong to the population of interest or ‘target’ population. Typically, data-sources are chosen so that they *only* observe members of the target population or are able to identify whether an individual is a member of the target population. However, this may not always be the case. For example, for the data that we introduce in Section 1.1, we are interested in the current number of people who inject drugs (PWID) in Scotland. Four data-sources are used to observe such individuals, including the Hepatitis C virus (HCV) diagnosis database. Individuals are identified as being PWID on this database, but their status as ‘current’ or ‘former’ injector is not recorded. Thus, this data-source records not only current PWID but also former PWID who do not belong to the target current PWID population of interest. A previous analysis of one year of these data by King *et al*. 11 simply discarded the HCV diagnosis database from the set of data-sources in the analysis. In this paper, we include the HCV diagnosis data-source and explicitly allow for the fact that not all individuals labelled as PWID by the HCV diagnosis database are members of the target population of current PWID. We now describe these data in more detail.

### 1.1. Data

In the last decade, data from multiple administrative data-sources have been repeatedly used to estimate the total number of current PWID in Scotland. We shall consider the data collected in the last three studies, corresponding to years 2003, 2006 and 2009. Four data-sources were used corresponding to social enquiry reports (S1), hospital records (S2), Scottish Drug Misuse Database (S3; SDMD) and HCV diagnosis database (S4). The HCV diagnosis database contains epidemiological data (including risk group and date of first diagnosis) on all individuals who have been newly diagnosed with HCV in Scotland. In addition, a range of covariates was recorded for each individual, including age (C1), gender (C2) and region (C3). For each study, age is categorised into two levels ( < 35 years, 35+ years). For 2003, region was categorised into the two levels, Greater Glasgow and Rest of Scotland, whereas in 2006 and 2009, the two levels were Greater Glasgow and Clyde, and Rest of Scotland. This change was to conform with the National Health Service board regions, which altered between the 2003 and 2006 studies. See King *et al*. 10,11 for further discussion of the data and for estimation of the current PWID in Scotland in 2003 and 2006, respectively. Data from the 2009 study have also recently been made available to the authors. See Table 1 for summary statistics of the data for all 3 years.

**Table 1:** Summary statistics for the data for years 2003, 2006 and 2009 showing the number of PWID observed in total and by each data-source.

		Social enquiry reports	Hospital records	SDMD	HCV diagnosis
Year	Total	S1	S2	S3	S4
2003	7201	1431	688	5151	761
2006	5670	901	953	3504	827
2009	4967	831	779	2946	888

SDMD, Scottish drug misuse database; HCV, Hepatitis C virus.

The number of individuals observed by each combination of data-sources and cross-classified across covariates can be presented in the form of an incomplete 2^7^ contingency table, with 8 ( = 2^3^) unknown cell counts. However, as discussed by 11, the PWID observed by the HCV diagnosis data-source are identified by their injecting drug use having been listed as an historical risk factor, which does not necessarily signify current injecting drug use. In other words, individuals observed by the HCV diagnosis data-source can be regarded as a combination of former and current PWID. King *et al*. 10 assumed that historical injecting drug use corresponded to current drug use for the 2003 data. For the 2006 data, because of an initiative to encourage former PWID, born in 1956–1975, to be tested for HCV 14–17, King *et al*. 11 conducted a sensitivity analysis by considering a further analysis excluding the HCV diagnosis data-source. Excluding the HCV diagnosis data-source resulted in a significant decrease in the estimated total population size for current PWID from a (model-averaged) posterior mean of 31 700 (to nearest 100), when using all four data-sources, to 25 000, when excluding the HCV diagnosis data-source.

### 1.2. Contributions and outline of paper

In this paper, we investigate, in further detail, the issue of individuals observed by the HCV diagnosis data-source including both target and non-target individuals. The main contribution of the paper is the development of a new modelling strategy to incorporate this complexity, without excluding the HCV diagnosis data-source, and hence, potentially, discarding a significant amount of information. The additional advantages of this strategy over the approach of 11, of removing the HCV diagnosis data-source, are that we are able to (i) estimate the proportion of individuals observed by the HCV diagnosis data-source who are current PWID and (ii) identify interactions between the HCV diagnosis data-source and the other data-sources and covariates. The results of both of these advantages are of interest to epidemiologists. Using this proposed modelling strategy, we provide, for the first time, estimates of the total population size of PWID in Scotland for 2009. We also revise the published estimates 10,11 of the total population size of PWID for 2003 and 2006, respectively, and for 2003, 2006 and 2009, we provide estimates of the proportion of individuals observed by the HCV diagnosis data-source who are currently PWID, for each age group.

The outline of the paper is as follows. Section 2 describes the new modelling strategy in detail and includes a simulation study to test the strategy's efficacy. In Section 3, we apply the strategy to the Scotland PWID multi-list data, for 2003, 2006 and 2009, to provide the aforementioned estimates. We end with Section 4 discussing future work and epidemiological conclusions of the results in Section 3.

## 2. Methodology

In this section, we discuss the assumptions relating to the modelling approach before describing the notation that will be used and the new model-fitting approach to deal with the HCV diagnosis data-source's potential for recording both former and current PWID.

### 2.1. Model formulation and assumptions

We assume that the HCV diagnosis data-source may record individuals from both current and former PWID (i.e. members of the target and non-target populations, respectively) but that each of the other data-sources only records current PWID. This implies that individuals recorded by the HCV diagnosis data-source and any other data-source are a current injector. Thus, only the observed cells corresponding to being recorded by *only* the HCV diagnosis data-source (i.e. observed by HCV diagnosis data-source and unobserved by all other data-sources) for each cross-classification of covariate values may contain non-target individuals. The corresponding cell entries can then be regarded as an upper bound on the true cell count (i.e. true number of current PWID observed by only the HCV diagnosis data-source), leading to *left censored* cells.

Notationally, we assume that there *n* cells in the incomplete contingency table, of which *n*_*U*_ are unobserved and *n*_*C*_ are censored. For each year of these data, *n* = 2^7^ = 128, *n*_*U*_ = 2^3^ = 8 (corresponding to being unobserved by each data-source for the 8 covariate cross-classifications) and *n*_*C*_ = 2^3^ = 8 (corresponding to being recorded by only the HCV diagnosis data-source for the 8 covariate cross-classifications). We label each cell from 1, … ,*n*, such that cells *i* = 1, … ,*n*_*C*_ correspond to the censored cells and cells *i* = *n*_*C*_ + 1, … ,*n*_*C*_ + *n*_*U*_ to the unobserved cells. For *i* = 1, … ,*n*, we let *y*_*i*_ denote the true number of current PWID in cell *i*. Further, we let 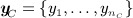 denote the (true) number of current PWID in the censored cells, 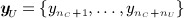 the number of current PWID in the unobserved cells and 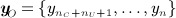 the observed cell entries for the uncensored cells. Next, we let 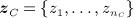 denote the observed cell entries for the censored cells. Thus, {***y***_*O*_,***z***_*C*_}are the observed data, whereas {***y***_*U*_,***y***_*C*_}are parameters to be estimated so that the total population size 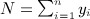 can be estimated. Finally, we let ***β*** denote the set of log-linear parameters in the model (we return to the issue of model uncertainty later).

We use a special case of a generalised linear model (GLM), where we assume that each (true) cell count, *y*_*i*_, has an independent Poisson distribution, that is, for *i* = 1, … ,*n*, 

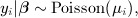
1 where *μ*_*i*_ has the form,

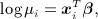
2 such that ***β*** denotes the (*q* + 1) × 1 vector of (identifiable) log-linear parameters, *β*_0_,*β*_1_, … ,*β*_*q*_, and ***x***_*i*_ the (*q* + 1) × 1 design vector relating to cell *i* = 1, … ,*n*. The term *β*_0_ corresponds to the intercept term, whereas *β*_1_, … ,*β*_*q*_ correspond to the main-effect and log-linear interaction terms. For parameter identifiability, we use the standard sum-to-zero constraints for the log-linear main-effect and interaction parameters. Because each data-source and covariate only has two levels, each element of ***x***_*i*_ is equal to ± 1. Finally, we let ***X*** denote the *n* × (*q* + 1) design matrix with *i*th row, ***x***_*i*_.

An alternative (and equivalent) model specification for ***y*** = {*y*_1_, … ,*y*_*n*_}is to set, 

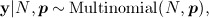


where the *i*th element of ***p*** is given by 

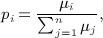
3 with *μ*_*i*_ given by Equation 2. This is the model specification considered by 5 and 6 (and subsequently 9–11) assuming no censored cells. This specification allows prior information on the total population size, *N*, to be directly incorporated through a prior distribution on *N*.

### 2.2. Posterior distribution

Given a log-linear model (in terms of the interaction terms present in the model), the posterior distribution of **y**_*U*_, **y**_*C*_ and all model parameters, denoted by ***θ***, given the observed data **y**_*O*_ and **z**_*C*_, can be written in the form, 

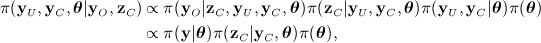
4 where ***y*** = {***y***_*O*_,***y***_*C*_,***y***_*U*_}, and *π*( · ) and *π*( · | · ) represent marginal and conditional probability density/mass functions, respectively. This result is derived because we assume that the observed cell entries **y**_*O*_ are independent of **z**_*C*_, **y**_*U*_ and **y**_*C*_, given ***θ***; and the observed censored cells **z**_*C*_ are independent of **y**_*U*_, given the log-linear parameters ***θ*** and true cell entries **y**_*C*_. The term *π*(**y** | ***θ***) corresponds to the complete data likelihood for the full set of cell entries, ***y***, as specified by Equation 1 and *π*(**z**_*C*_ | **y**_*C*_,***θ***) the distribution of the observed upper bound for the censored cells, given the log-linear parameters and true cell entries. For these data, we assume non-informative censoring. In other words, *z*_*i*_ | *y*_*i*_ ∼ *U*[*y*_*i*_, ∞ ), independently, for *i* = 1, … ,*n*_*C*_ (and *π*(**z**_*C*_ | **y**_*C*_,***θ***) = *π*(**z**_*C*_ | **y**_*C*_)). In Section 4, we discuss an alternative distribution for *z*_*i*_, which can be used to incorporate individual prior information on the proportion of individuals observed by the HCV diagnosis data-source who are current PWID.

To allow for additional model uncertainty, in terms of the log-linear interaction terms that are present in the model, we extend the posterior distribution given in Equation 5. We treat the model indicator as a discrete parameter and form the joint posterior distribution over both parameters and model space. Let 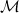 denote the set of models we wish to consider and let 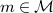 denote the model indicator and let ***θ***_*m*_ denote the parameters present within model 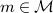. The joint posterior distribution is given by 

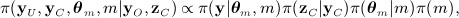
5 where *π*(*m*) denotes the prior model probability for model 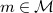. In this paper, we assume that 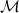 consists of hierarchical log-linear models 18. Hierarchical models include, as subsets, the classes of graphical and decomposable models. These models adhere to the principle of marginality, that is, to have a higher-order interaction, we need to have all constituent lower-order interactions. Typically, the simplest (or minimal) model we often consider is the independence model. The independence model has main-effect terms for all of the data-sources and covariates but no interaction terms. We may also wish to specify a maximal model, that is, the most complex model we wish to consider, which is performed by specifying the highest-order interaction.

### 2.3. Prior distribution

Within the Bayesian approach, we can incorporate any available prior information on the model parameters, ***θ***_*m*_ and *m*, into the analysis via the prior distributions, *π*(***θ***_*m*_ | *m*) and *π*(*m*). However, we assume there is weak prior information on the model parameters and so need to specify a prior distribution, which reflects this position. In general, care must be taken when specifying prior distributions on the model parameters under weak prior information in the case of model uncertainty due to Lindley's paradox 19 where the posterior model probabilities are sensitive to the scale of the prior variance for each ***β***_*m*_. To counter this issue, we use the idea of ‘default prior distributions’ 20, which aim to provide consistent amounts of prior information under each model, that is, they are compatible across models. Specifically, we use the generalised hyper g-prior (GHGP), which is an example of a default prior distribution proposed by Sabanes-Bové and Held 21 for GLMs. To describe this prior for ***β***_*m*_, we drop the subscript *m*, for notational simplicity. Because the intercept, *β*_0_, is present in all models, we assume *π*(*β*_0_) ∝ 1. Let the matrix **Y** be formed from **X** by removing the first column of ones corresponding to *β*_0_. The GHGP for the remaining elements, ***α*** = (*β*_1_, … ,*β*_*q*_), of ***β*** is given by ***α*** | *σ*^2^ ∼ N (**0**,*σ*^2^*n***Y**^*T*^**Y**), where *σ*^2^ > 0 is an unknown hyperparameter. The GHGP extends the Zellner g-prior 22 for linear models to the wider class of GLMs. The basic idea is that GHGP is the posterior distribution resulting from an analysis under a locally uniform prior distribution for ***α*** and an imaginary sample of size 1 / *σ*^2^, which is referred to as the ‘prior sample size’ by 18. The unit information prior for GLMs 23, which is another default prior applicable in this case, is formed by setting *σ*^2^ = 1, thus giving a prior sample size of one. This is intuitively appealing because the amount of information the prior provides is directly interpretable. Sabanes-Bové and Held 21 extended this by letting 1 / *σ*^2^ be unknown by assuming a gamma hyperprior distribution with parameters *a* / 2 and *b* / 2, so that, on the inverse scale 

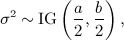


where IG denotes the inverse-gamma distribution. We specify *a* = *b* = 10^ − 3^ so that the hyperprior on 1 / *σ*^2^ is vague but centred at one. However, in Section 3, we investigate the sensitivity of the resulting analysis to these values of *a* and *b*. The inverse-gamma hyperprior distribution assumed earlier also has the minor computational advantage of being conditionally conjugate (see step 1 in Section 2.4) meaning the full conditional distribution of *σ*^2^ is also an inverse-gamma distribution. Because, in our case, all of the data-sources and covariates have two levels, under sum-to-zero constraints, the GHGP corresponds to the elements of ***α*** being independent with variance given by *σ*^2^.

Finally, we specify a uniform prior over all admissible models, that is, 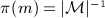, where 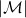 is the number of admissible models. However, the computational methods will perform in a similar fashion for any prior over the model space. The set of parameters for model *m* is given by ***θ***_*m*_ = {***β***_*m*_,*σ*^2^}.

The joint posterior distribution of **y**_*U*_, **y**_*C*_, ***θ***_*m*_ and *m* under the Poisson specification is identical to that obtained using the multinomial model formulation when *π*(*N*) ∝ *N*^ − 1^, corresponding to Jeffreys’ prior 5, assuming identical priors on all other parameters and over the model space. This result extends a result of Forster 24 for complete contingency tables and is proved in Section 1 of the Supporting information. Thus, in the presence of no expert prior information (and the Uniform prior on the intercept term for the Poisson formulation or Jeffreys’ prior on the total population for the multinomial formulation), the different model specifications are equivalent. However, the Poisson log-linear model specification is a special case of a GLM, which in turn permits the application of an efficient general computational algorithm (Section 2.4).

The aim of this paper is to consider the impact of censored contingency cells, and so, to maximise consistency across years, we specify the same default prior from above on the parameters for each dataset. The previous analyses of the Scotland PWID multi-list data have used the alternative multinomial model specification in order to specify an informative prior on the total population size, *N* (obtained by combining the estimated number of injecting drug related death rates with an estimated associated death rate). Thus, the posterior distributions for the total population size ignoring the censored cells for 2003 and 2006 will be slightly different to those published in 10 and 11, respectively, because of this different prior specification.

The priors we have specified earlier are not conjugate. In fact, for hierarchical log-linear models, conjugate priors do not exist. This means it is necessary to employ the computational methods, described in Section 2.4, to evaluate the marginal posterior distribution of the total population size, *N*. Under the multinomial model formulation, and for the subset of decomposable models, Madigan and York 5 use a hyper-Dirichlet distribution 25 on ***p***, which gives a closed form expression for the marginal posterior distribution of the total population size. However, as Dellaportas and Forster 18 noted, we should not restrict ourselves to a less flexible class of models for solely computational reasons. In Section 2.5, in a small simulation study, we compare our approach using hierarchical models to the approach of 5 using decomposable models.

### 2.4. Computation

In this section, we describe the computational algorithm used to generate an MCMC sample from the posterior distribution of ***y***_*U*_, ***y***_*C*_, ***θ***_*m*_ and *m*, given by Equation 5. The algorithm employed is an example of data-augmentation and, as such, can be viewed as iterative multiple imputation 3. The algorithm has two steps:

1. Step 1 Conditional on the current values of the unknown and censored cell counts, ***y***_*U*_ and ***y***_*C*_, update the parameters and model, ***θ***_*m*_ and *m*.
2. Step 2 Conditional on the current parameters and model, ***θ***_*m*_ and *m*, update the unknown and censored cell counts, ***y***_*U*_ and ***y***_*C*_.

We consider each step in turn.

#### Step 1: Updating the parameter and model

The log-linear parameters, ***β***_*m*_, and model, *m*, are updated using the reversible jump (RJ) algorithm 26. Automated RJ algorithms have been proposed for arbitrary models (see, for example, 27–29) and specifically for GLMs (see, for example, 18,30,31). We use the implementation of 31 in which it was shown that the proposed implementation outperformed that of 18 among other implementations. The technical details are provided in Section 3 of the Supporting information. This implementation is appropriate where we have specified prior distributions on the model parameters that reflect weak prior information. Within this RJ algorithm, we also need to specify the ‘null move’, that is, where *m* remains unchanged, and we update only the log-linear parameters, ***β***_*m*_. Previous approaches, including the study of King and Brooks 6, typically consider a single-update random walk Metropolis–Hastings algorithm and use pilot-tuning to obtain the proposal variances 32. However, the optimal proposal variance will often depend on the current model *m*. We use an alternative algorithm that utilises the iterated weighted least squares implementation of the Metropolis–Hastings algorithm for GLMs 33. This algorithm is described for log-linear models in Section 2 of the Supporting information.

Now consider the hyperparameter, *σ*^2^. The inverse gamma prior distribution for *σ*^2^ is conditionally conjugate so that we update the parameter using the closed form of the full conditional distribution, that is, 

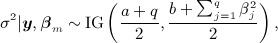
6 where ***β***_*m*_ = (*β*_1_, … ,*β*_*q*_).

#### Step 2: Updating the cell entries

To update the unobserved cell entries 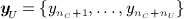, the full conditional distribution is simply the corresponding Poisson distribution, given in Equation 1, that is, *y*_*i*_ | ***β***_*m*_ ∼ Poisson(*μ*_*i*_). Similarly, to update the true cell entries for the censored cells, 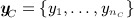, the full posterior conditional distribution is simply a truncated Poisson distribution, such that *y*_*i*_ | ***β***_*m*_,*z*_*i*_ ∼ Poisson(*μ*_*i*_)*I*(*y*_*i*_ *⩽ z*_*i*_), where *I* denotes the indicator function.

The computational approach described in this section is implemented in the R 34 package conting 35, which is available to download from the Comprehensive R Archive Network. This package includes the data from 2006.

### 2.5. Simulation study

In this section, we conduct a small simulation study to assess the efficacy of the proposed modelling strategy and to compare this against other methods. The simulation study attempts to match the observed properties of the Scotland PWID multi-list data introduced in Section 1.1 and its inferred properties from Section 3.

The simulation study is set up as follows. We assume there are four data-sources: S1, S2, S3 and S4, and that S4 is the censored data-source observing a mixture of individuals from the target and non-target populations. For simplicity, we assume that there are no covariates. This means that there are 16 cells in the table. One cell count, *y*_*U*_, is completely unknown; one cell count, *y*_*C*_, is unknown but bounded from above by *z*_*C*_; and the remaining 14 cell counts, **y**_*O*_, are observed. We consider three different true total population sizes *N* = 10000,15000 and 20000. Additionally, we consider two different forms for the true data-generating model: (i) a model with non-zero interactions for S1 : S2, S1 : S4 and S2 : S4 and (ii) a model with a non-zero interaction for S1 : S2. Note that model (ii) is decomposable but model (i) is not decomposable (as it is not even graphical). Table 2 shows the true values of ***β*** under each of the true data-generating models. These values were chosen so that the simulated data were consistent with the observed Scotland PWID multi-list data. For example, the negative values of the true parameters associated with the main-effect terms mean that the probability of observing an individual is less than that of not observing an individual.

**Table 2:** True values of the model parameters for the two different true data-generating models considered in the simulation study in Section 2.5.

Term	Parameter	Value under model (i)	Value under model (ii)
S1	*β*_1_	− 0.75	− 0.75
S2	*β*_2_	− 0.75	− 0.75
S3	*β*_3_	− 0.75	− 0.75
S4	*β*_4_	− 0.75	− 0.75
S1 : S2	*β*_5_	0.25	0.25
S1 : S3	*β*_6_	0.00	0.00
S1 : S4	*β*_7_	0.25	0.00
S2 : S3	*β*_8_	0.00	0.00
S2 : S4	*β*_9_	0.25	0.00
S3 : S4	*β*_10_	0.00	0.00

Model (i) has three non-zero interactions and model (ii) has one.

Using the parameter values in Table 2 and each value of *N*, we generate the true cell counts from the multinomial distribution. We remove the cell count, *y*_*U*_, corresponding to not being observed by any of the data-sources. We also replace *y*_*C*_ (the cell count corresponding to just being observed by S4) by *z*_*C*_, where *z*_*C*_ is generated from a negative binomial distribution (under the parameterisation of 36) with number of successes *y*_*C*_ and probability 2 / 3 (so as to be consistent with the inferred properties from the observed data for PWID in Scotland). For each true data-generating model and each true total population size, we generate 1000 datasets using the aforementioned procedure. We analyse each dataset using the following four methods.

INC-C The proposed modelling strategy, described in Sections 2.1 to 2.4, of incorporating the censoring mechanism, using hierarchical models.
REM-C The approach of 11 of removing the censored data-source and employing only data-sources S1, S2 and S3, using hierarchical models.
IGN-C The approach of 10 of ignoring the censoring problem, using hierarchical models.
MY-C The approach of 5, using decomposable models, extended to incorporate the censoring mechanism.

Note that the MY-C method of 5 does not immediately provide a solution to the censoring problem. However, we adopt our proposed approach of sampling the true cell count, *y*_*C*_, in the censored cell from a truncated posterior predictive distribution. Also, note that, to apply the approach of 5, we need to specify a prior distribution for the total population size, *N*. To maintain consistency with the three other approaches listed earlier, we assume Jeffreys’ prior for *N*, that is, *π*(*N*) ∝ *N*^ − 1^ (Section 2.3).

Before we present the results of the simulation study, we briefly contrast the relative advantages and disadvantages of the four methods. The REM-C and IGN-C methods are the simplest approaches because we do not model the censoring by either removing the data-source or ignoring the problem altogether, respectively. The REM-C method will not be able to evaluate the posterior distribution of the proportion, *φ*, of individuals observed by the S4 data-source who are actually members of the target population or identify interactions involving the S4 data-source. Additionally, for this method, by removing the censored data-source, we are discarding information. The INC-C and MY-C methods actually model the censoring mechanism and will be able to provide a posterior distribution of *φ*. Additionally, these two methods can identify interactions involving the S4 data-source. Finally, the INC-C method has the advantage over the MY-C method of considering the much more flexible class of hierarchical models. However, this advantage comes at the price of requiring more complex computational methods to evaluate the resulting posterior distributions.

Table 3 shows the coverage rates and mean lengths (relative to the INC-C method) for 95% highest posterior density intervals (HPDIs) for the total population size, *N*, and *φ*. These rates and lengths, averaged over the 1000 datasets, are given for each of the three true values for *N*, each of the two true data-generating models and each of the four methods. An entry of NA indicates that this method cannot estimate the value of *φ*. Note that the true value of *φ* depends on the true cell counts generated for each individual dataset.

**Table 3:** Coverage rates and mean lengths (relative to the INC-C method) of 95% HPDIs from the simulation study in Section 2.5 for the total population size, *N*, and the proportion, *φ*, of individuals observed by the S4 data-source who are members of the target population.

True total population size	Method	Coverage rate (%)				Relative mean length			
		Model (i)		Model (ii)		Model (i)		Model (ii)	
		*N*	*φ*	*N*	*φ*	*N*	*φ*	*N*	*φ*
10 000	INC-C	93	93	93	95	1.00	1.00	1.00	1.00
	REM-C	93	NA	94	NA	1.00	NA	1.02	NA
	IGN-C	35	NA	2	NA	1.37	NA	1.06	NA
	MY-C	90	88	93	92	1.02	0.98	1.12	1.03
15 000	INC-C	92	94	94	96	1.00	1.00	1.00	1.00
	REM-C	92	NA	94	NA	1.00	NA	1.01	NA
	IGN-C	26	NA	2	NA	1.49	NA	1.04	NA
	MY-C	89	87	92	91	1.03	1.01	1.13	1.06
20 000	INC-C	93	96	95	96	1.00	1.00	1.00	1.00
	REM-C	94	NA	94	NA	1.00	NA	1.02	NA
	IGN-C	21	NA	1	NA	1.57	NA	1.03	NA
	MY-C	89	87	92	91	1.06	1.01	1.18	1.09

The coverage rates and relative mean lengths are given for each true total population size (10 000, 15 000 and 20 000), each true data-generating model (non-zero interactions for (i) S1:S2, S1:S4 and S2:S4 and (ii) S1:S2) and each of the four methods. Coverage rate refers to the proportion of intervals that contain the true value of the parameter. The mean length refers to the mean of the difference between the lower and upper bounds of each interval. An entry of NA indicates that an estimate of this parameter is not available under this method.

In terms of coverage, the IGN-C method performs poorly. Further investigation shows that the true value of *N* was less than the lower bound of the interval under the IGN-C method for every occurrence of the interval not covering *N*. This indicates that the IGN-C method over-estimates the population size under the chosen two true data-generating models. The remaining methods, INC-C, REM-C and MY-C, all perform fairly well. As expected, the MY-C method performs in a more satisfactory manner when the true data-generating model is a decomposable model, that is, for model (ii). The INC-C and REM-C methods perform very similarly. However, the INC-C method has the advantage of being able to estimate *φ*.

In terms of the mean lengths of the intervals, the INC-C and REM-C methods perform similarly again with, typically, the INC-C intervals being slightly shorter, that is, more precise. This supports our intuition, that by removing the censored data-source, we would be discarding information. However, the actual different in mean lengths between the INC-C and REM-C methods is small. Typically, the MY-C intervals are slightly wider than the INC-C and REM-C intervals, and this difference is more obvious under the decomposable model (ii).

We further compare the INC-C, REM-C and IGN-C methods using the Scotland PWID multi-list data in Section 3.

## 3. Applying the proposed modelling strategy to the Scotland people who inject drugs multi-list data in 2003, 2006 and 2009

We apply the proposed methodology (INC-C) from Section 2 to the Scotland PWID multi-list data for 2003, 2006 and 2009. For comparison, we also conduct two additional analyses for each dataset: (i) the approach of 11 of removing the HCV diagnosis data-source (REM-C) and (ii) the approach of 10 of ignoring the censoring problem (IGN-C). For all analyses, we assume that the maximal model consists of the model with all two-way interactions present but no higher-order interactions. This appears to be sufficient to produce an adequate overall model according to the Pearson- *χ*^2^ statistic and its associated Bayesian *p*-value (Section 3.1). In a similar analysis of multi-list data from England, King *et al*. 12 found that there was negligible difference in overall population estimates when including higher-order interactions. For the INC-C and IGN-C (four data-sources) analyses, where the maximal model is as specified earlier, there will be over two million possible models in 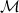. For the REM-C (three data-sources) analyses, there will be over 32 000 models. Such large model spaces require large MCMC sample sizes. For each analysis, the MCMC algorithm was run for two million iterations. MCMC convergence was assessed using trace plots 19 and Geweke's diagnostic 37 based on the samples from the posterior distribution of parameters corresponding to the intercept and main effects. In all cases, there was no evidence of a lack of convergence with the aforementioned MCMC sample size. The exception to this is for 2009 under the INC-C and REM-C methods. This is due to the posterior distribution for the total population size being significantly bi-modal with the probabilities of the two modes being comparable. It was decided to run these two chains for five million iterations to obtain more precise estimates of the relative probabilities of these two modes. The issue of bi-modality of the posterior distribution of the total population size is discussed in more detail in Section 3.2. Using the R package conting, on a desktop PC with a quad core 3.20 GHz processor and 16 Gb of memory, two million iterations of the algorithm for INC-C analyses took between 330 and 340 min, for REM-C between 255 and 265 min and for IGN-C between 315 and 325 min. The algorithms with five million iterations took proportionately longer. The acceptance rates for the within-model Metropolis–Hastings moves were for INC-C, between 75% and 80%; for REM-C, between 84% and 86%; and for IGN-C, between 74% and 79%. The acceptance rates for the between-model RJ moves were for INC-C, between 6% and 8%; for REM-C, between 4% and 6%; and for IGN-C, between 5% and 8%. For all MCMC chains, the first 10% of the iterations was discarded as burn-in.

### 3.1. Assessing model adequacy

In order to assess overall goodness-of-fit of the four data-source analyses, we calculate the (model-averaged) Bayesian *p*-value 3 using the Pearson- *χ*^2^ statistic on the observed cell entries, ***y***_*O*_, as the discrepancy function. We take a pragmatic approach whereby a Bayesian *p*-value in the interval (0.05,0.95) indicates no lack of a goodness-of-fit to the data of the model-averaging approach. For the INC-C analyses, we obtain Bayesian *p*-values of 0.15, 0.44 and 0.30 for years 2003, 2006 and 2009, respectively; for the IGN-C analyses, values of 0.09, 0.27 and 0.08, respectively; and for the REM-C analyses, values of 0.06, 0.28 and 0.12, respectively. Thus, for all analyses, the Bayesian *p*-value suggested an adequate goodness-of-fit for each method. Therefore, these do not suggest that the IGN-C modelling approach that ignores censoring is inadequate, but these assessments use the same false assumption as the model does, that is, that all individuals observed by the HCV diagnosis data-source are current PWID.

### 3.2. Population estimates

**Figure 1:**
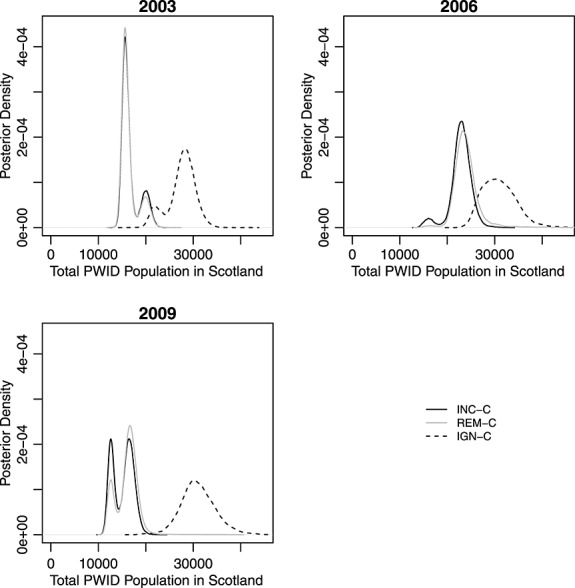
Plots of the posterior density for the total number of people who inject drugs (PWID) in each year for the INC-C, REM-C and IGN-C methods.

The corresponding posterior estimates for the total population size for current PWID are provided in Table 4 for each analysis, and posterior marginal distributions are displayed graphically in Figure 1. Clearly, the estimates of the total population sizes are significantly larger when analysing the contingency table having ignored the censoring (IGN-C) of the observed cells than when formally modelling the censored cells (INC-C) or when removing the HCV diagnosis data-source altogether (REM-C). The INC-C and REM-C analyses provide very similar posterior estimates. Both of these outcomes agree with the conclusions from the simulation study in Section 2.5.

**Table 4:** Posterior mean and 95% highest posterior density intervals for the total PWID population size in Scotland (to nearest 100) for the years 2003, 2006 and 2009 using the INC-C, REM-C and IGN-C methods.

Year	INC-C		REM-C		IGN-C	
	Posterior mean	95% HPDI	Posterior mean	95% HDPI	Posterior mean	95% HDPI
2003	16 700	(14 300, 20 900)	16 500	(14 200, 20 800)	27 500	(20 700, 32 300)
2006	22 900	(16 300, 27 000)	24 000	(19 500, 29 700)	31 000	(24 600, 37 700)
2009	15 200	(11 500, 18 600)	16 000	(11 500, 19 400)	31 000	(24 000, 38 900)

HPDI, highest posterior density interval.

Figure 1 shows that, for 2003, the posterior distributions all appear to be bi-modal; and in 2009, so too do the posterior distributions for the REM-C and INC-C analyses (with minor bi-modality in 2006 for the same analyses). This bi-modality is a result of averaging across different log-linear models. A range of models is identified with positive posterior support, with some of these models providing reasonably different estimates of the total population size, leading to the separate smaller sub-modes. In particular, the separate modes for each year correspond to the inclusion/exclusion of the S1 × S3 (social and SDMD) interaction. For example, for the INC-C analysis for the 2009 data, the posterior probability of including this interaction is 0.62, contributing to the upper mode of the posterior distribution (with higher posterior estimates for population size when the interaction is present, because the interaction has a positive posterior mean). Typically, this interaction (when present) has a positive posterior mean, with the exception of the 2003 data for INC-C and REM-C. For these analyses, the lower mode corresponds to the inclusion of this parameter (posterior probability of presence: 0.80 and 0.83 for INC-C and REM-C, respectively).

Table 5 shows posterior estimates of the total population size (for all three methods) for each age group. Again, the posterior estimates under the INC-C and REM-C methods are very similar. A peak in 2006 is now clear for the older age group with an approximate doubling in the number of current PWID between 2003 and 2006 but thereafter a significant fall of the posterior mean from 7800 to 4800 in 2009. For the younger age group, there appears to be a corresponding fall between 2006 and 2009 (posterior mean from 15 100 to 10 400) without having seen such a pronounced increase between 2003 and 2006.

**Table 5:** Posterior mean and 95% highest posterior density intervals for the total population size (for INC-C, REM-C and IGN-C) and the proportion of individuals observed by the Hepatitis C virus diagnosis data-source who are current people who inject drugs (for INC-C). These posterior estimates are given for each year and for each age group.

Year	INC-C			REM-C		IGN-C	
	Posterior mean	Posterior mean		Posterior mean		Posterior mean	
	(95% HPDI)	(95% HPDI)		(95% HPDI)		(95% HPDI)	
	for proportion	for total population		for total population		for total population	
< 35 years							
2003	0.62 (0.49, 0.78)	12800	(10900, 16000)	12700	(10800, 16000)	19900	(15100, 23300)
2006	0.68 (0.50, 0.81)	15100	(10800, 17900)	15900	(12700, 19600)	18900	(15000, 23100)
2009	0.58 (0.41, 0.73)	10400	(7800, 12800)	11000	(7800, 13400)	17000	(13100, 21400)
35 + years							
2003	0.46 (0.32, 0.63)	3800	(2800, 5300)	3800	(2700, 5200)	7600	(5500, 9200)
2006	0.51 (0.34, 0.68)	7800	(5300, 10100)	8100	(6000, 10600)	12000	(9000, 15200)
2009	0.22 (0.13, 0.31)	4800	(3600, 6000)	5100	(3600, 6200)	14000	(10200, 17900)

HPDI, highest posterior density interval.

### 3.3. Interactions

Table 6 provides the corresponding posterior (marginal) probabilities for each possible interaction being present in the model for three methods. Note that Table S1 in Section 4 of the Supporting information shows the corresponding posterior mean of the log-linear parameters for these interactions. Within years, the posterior probabilities for the interaction terms are generally consistent in interpretation except for S1 × S2 (social and hospital) and S2 × S3 (hospital and SDMD) in 2003; S2 × S3 (hospital and SDMD) and S4 × Region (HCV and region) in 2006; S1 × S2 (social and hospital), S2 × S3 (hospital and SDMD), S3 × Age (SDMD and age) and S4 × Region (HCV and region) in 2009.

**Table 6:** The marginal posterior probability for each two-way log-linear interaction term for the INC-C, REM-C and IGN-C methods for the years 2003, 2006 and 2009.

Interaction	2003			2006			2009		
	INC-C	REM-C	IGN-C	INC-C	REM-C	IGN-C	INC-C	REM-C	IGN-C
S1 × S2	0.10	0.10	0.92	0.14	0.10	0.08	0.11	0.16	0.99
S1 × S3	0.80	0.83	0.85	0.94	0.99	1.00	0.62	0.79	1.00
S1 × S4	0.09	NA	0.15	0.12	NA	0.08	0.33	NA	0.14
S2 × S3	0.24	0.22	1.00	0.11	0.11	0.65	0.40	0.27	0.99
S2 × S4	1.00	NA	1.00	1.00	NA	1.00	0.94	NA	0.57
S3 × S4	0.11	NA	0.17	0.17	NA	0.09	0.12	NA	0.12
S1 × Age	1.00	1.00	1.00	1.00	1.00	1.00	0.69	0.67	1.00
S2 × Age	0.44	0.48	0.07	0.99	0.98	0.92	1.00	1.00	1.00
S3 × Age	0.80	0.76	1.00	1.00	1.00	1.00	0.16	0.16	1.00
S4 × Age	0.09	NA	0.12	0.07	NA	0.15	0.32	NA	0.22
S1 × Sex	1.00	1.00	1.00	0.08	0.08	0.13	0.07	0.07	0.13
S2 × Sex	0.07	0.07	0.06	1.00	1.00	1.00	1.00	1.00	1.00
S3 × Sex	0.08	0.08	0.07	0.23	0.05	0.06	0.04	0.04	0.04
S4 × Sex	0.11	NA	0.06	0.08	NA	0.13	0.07	NA	0.11
S1 × Region	0.93	0.93	0.98	0.21	0.20	0.47	0.07	0.07	0.20
S2 × Region	1.00	1.00	1.00	0.05	0.06	0.27	0.04	0.05	0.14
S3 × Region	0.06	0.07	0.07	1.00	1.00	1.00	1.00	1.00	1.00
S4 × Region	0.98	NA	1.00	0.09	NA	0.82	0.17	NA	1.00
Age × Sex	1.00	1.00	1.00	1.00	1.00	1.00	1.00	1.00	1.00
Age × Region	1.00	1.00	1.00	1.00	1.00	1.00	1.00	1.00	1.00
Sex × Region	0.04	0.04	0.02	0.04	0.04	0.04	0.03	0.04	0.03

The data-sources are labelled as S1, social enquiry reports; S2, hospital records; S3, Scottish drug misuse database (SDMD); and S4, HCV diagnosis data-source. An NA indicates that this interaction cannot be identified with the REM-C method.

For 2003 and 2006, the IGN-C analysis lends additional support for a further one interaction terms being present (i.e. posterior model probability > 0.75); for 2009, a further six interaction terms have strong positive support, whereas the interaction term S2 × S4 (hospital and HCV) loses posterior support. For the interactions identified under the IGN-C method, the sign of the posterior mean of the data-source × data-source interactions are all positive, which leads to the increased population estimates.

Finally, for all interactions where it is deemed that there is positive evidence that they are present, except the S1 × S3 (social and SDMD) interaction discussed earlier, the sign of the posterior means of the interactions were consistent across all years and analyses.

### 3.4. Proportion of individuals observed by the Hepatitis C virus diagnosis data-source who are current people who inject drugs

As stated, an advantage of the INC-C analysis over the REM-C method is that it allows us now to consider the proportion of individuals identified by the HCV diagnosis data-source as having a risk factor of injecting who are actually current PWID (i.e. members of the target population).

Because of the initiative (Section 1.1) to encourage former PWID, born in 1956–1975, to be tested for HCV diagnosis, we consider the posterior distribution of the proportion of current PWID for the two different age groups. Table 5 shows the posterior mean and 95% HPDIs of this proportion for both age groups for each year. Comparing the two age groups, it appears that the proportion of older individuals on the HCV diagnosis data-source who are current PWID is lower than for younger individuals. The largest discrepancy between the age groups is in 2009 where there seems to have been a significant decrease in the proportion for the older age group compared with 2003 and 2006. This can be compared with the younger age group, where the proportion appears relatively stable over time. This would support the hypothesis that, in the older age group, there has been an increase in the number of former PWID being tested for HCV diagnosis and hence being identified on the HCV diagnosis data-source.

### 3.5. Prior sensitivity

To investigate the sensitivity of the analysis to the values of the hyperparameters, *a* and *b*, which specify the prior distribution for *σ*^2^ (Section 2.3), we repeat the INC-C analysis for a range of values of these hyperparameters. To do this, we fix *a* = 0.001 and vary *b* according to the expectation of the prior sample size 1 / *σ*^2^. We choose *b* = 0.004,0.002 and 0.0005 corresponding to expectations of the prior sample size of 4, 2 and 0.5, respectively. Additionally, we also use the Gelman prior 38, where a uniform prior (with some suitable large upper bound) is assumed for *σ* so that *a* = − 1 and *b* = 0 in the full conditional distribution of *σ*^2^ given by 8. We compare the resulting posterior mean of the total population size under each of the four priors to the corresponding posterior mean under the prior specified in Section 2.3 where *a* = 0.001 and *b* = 0.001, for each year. In each case, the resulting posterior mean is within 4% of the corresponding mean for when *a* = 0.001 and *b* = 0.001. Note that the posterior means under the four priors are given in Table S1 of Section 4 of the Supporting information, along with 95% HPDIs.

## 4. Discussion

Within standard capture-recapture studies, it is assumed that all individuals that are observed by the data-sources are members of the target population of interest. If this is not the case, the results obtained can lead to biased population estimates. For the data studied within this analysis, the HCV diagnosis database identified as PWID those who had listed injecting as their HCV risk factor, thus including former PWID in addition to the target population of current PWID. In such instances, one possibility is simply to remove such data-sources from the capture-recapture analyses. However, this may remove a significant amount of information that is contained within the data-source(s). In this paper, we have accounted for the possibility of censoring occurring for a single data-source (i.e. a single data-source may observe non-target individuals) by explicitly accounting for the censoring within the modelling structure for the HCV diagnosis database.

In particular, we considered estimating the total number of current PWID in Scotland in the years 2003, 2006 and 2009 allowing for censoring for the HCV diagnosis database and compared this with the analyses that ignored the potential censoring issue or removed the HCV diagnosis database and considered only three data-sources. Consistently, the analyses that ignored the issue of the HCV diagnosis database observing a mixture of former and current PWID led to significantly larger estimates for the total population size than the other two approaches that accounted for this (either by directly modelling the censoring or simply removing the given data-source). This conclusion was confirmed by the simulation study in Section 2.5.

The approach of removing the HCV diagnosis data-source produced remarkably similar posterior estimates to that of incorporating the censoring. However, the analysis incorporating censoring allowed us to estimate the proportion of those present on the HCV diagnosis database with a history of drug use who are actually current PWID and identify interactions involving the HCV diagnosis data-source. Both of these are of interest to epidemiologists.

We divide the remainder of this concluding section into two parts. In Section 4.1, we discuss ways in which the proposed modelling approach that incorporates censoring can be extended by considering a larger model space, 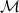, and by incorporating informative censoring. In Section 4.2, we discuss the epidemiological impact of the most up-to-date estimates of the total population size of current PWID in Scotland in 2009, as well as the revised estimates for 2003 and 2006. Additionally, we also discuss the impact of the estimates of the proportion of those present on the HCV diagnosis database who are current PWID.

### 4.1. Extensions to the proposed modelling approach

In this section, we discuss two ways in which the modelling approach, given in Section 2, can be extended. First, in Section 3, the maximal model was specified as the model containing all of the two-way interactions. This gave sufficient complexity to obtain an adequate model according to the Bayesian *p*-value. This may not always be the case and we may need to consider a larger set of models, 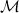, by considering higher-order interactions. In this case, a uniform prior over the model space, that is, 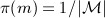, is deceptive. When the maximal model only contains two-way interactions and we have assumed a uniform prior on the model space, the prior probability of each two-way interaction is 1/2. However, this will not be the case when we consider higher-order interactions, where the prior probability of each two-way interaction will be greater than 1/2. We could refine our prior model probabilities in this case (39). The MCMC algorithm described in Section 2.4 would remain unchanged and should still perform satisfactorily because the RJ implementation employed is based on local proposals and will be unaffected by changes in the prior model probabilities.

Second, in this paper, we considered uninformative censoring, that is, the distribution of the upper bound, *z*_*i*_, on the censored cell count, *y*_*i*_, for each cross-classification of covariates, is uniform, namely, 

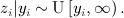


We may actually have some prior information on the proportion, *ψ*_*i*_, of individuals, within each cross-classification, *i*, of covariates, observed *solely* by the HCV diagnosis data-source who are currently PWID. One way to incorporate this is to assume that 

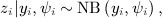


where NB denotes the negative binomial distribution 36. Under this parameterisation of the negative binomial distribution, *z*_*i*_ is the random variable of the number of trials given *y*_*i*_ successes and a probability of success *ψ*_*i*_. In this context, the number of trials is the number of individuals observed solely by the HCV diagnosis data-source and the number of ‘successes’ is the number of current PWID. The prior information on *ψ*_*i*_ may be in the form of a point estimate or encoded in a prior distribution, for example, a beta distribution.

### 4.2. Epidemiological impact

We found that Scotland's number of current PWID had risen between 2003 and 2006 but decreased by 2009. This is good news in public health terms but celebration should be cautious because cessation from injecting need not mean that the individuals concerned have ceased opiate dependency. The dramatic decrease in Scotland's number of current PWID between 2006 and 2009, including among those aged 35 +  years (Table 5), means that there may have been a corresponding increase in the number of those individuals receiving methadone as an opiate substitution therapy. Scotland's increase in older methadone-related deaths in 2011 has been much debated since their publication in mid-August 2012 40. In particular, the Scottish Daily Record 41 has called for much better information on the age-distribution of methadone-clients and to know whether methadone-related deaths occurred in those to whom methadone was prescribed or diverted. Answers to these questions are all the more important in view of our results, which strongly suggest that Scotland has reversed the rise in current PWID that had been apparent in 2006, and that the decrease has come about in both age-groups, although probably for different reasons. In the younger age group, aversion to injecting, and thereby reduction of new recruits into injecting 42, may have played a part in addition to cessation from injecting drug use as a consequence of being recruited into opiate substitution therapy. In the older age group, diagnosis of HCV carriage while still actively injecting may have been a reason to try to cease injecting both so that antiviral clearance of HCV carriage would not be undermined by re-infection and also, altruistically, to avoid onward injection-related HCV transmission. In the older age group, higher age-related risk of drugs-related death per 100 current PWID may have contributed, and for those who continued to inject while also receiving opiate substitution therapy, the question of higher risk of methadone-related death in older individuals is, as yet, unresolved.

Table 5 provides estimates, by age group, of the proportion of new HCV diagnosed individuals with historical injecting drug use who were current PWID at the time of their HCV diagnosis: around three-fifths of those under 35 years of age but a substantially lower proportion, even in 2003 (around 40%), of those aged 35 +  years, and by 2009, the proportion had decreased further to 23%.

Finally, the problem of Scotland's high drug related death rates 43 deepens because, when censoring is taken into account, we reduce Scotland's estimated number of current PWID in 2003–2009, and hence, because the numerator of opiate-related drug related deaths (DRDs) is unchanged, we revise upwards posterior estimates of opiate-related DRDs per 100 current PWID, in the older age group in particular.

In summary, Scotland, between 2006 and 2009, has both encouraged older former PWID to be HCV-diagnosed and recorded reductions in its number of current PWID in both age groups ( < 35 years, 35 +  years).

## Supporting Information

Supporting info item
