# Supplementary material for: Incomplete contingency tables with censored cells with application to estimating the number of people who inject drugs in Scotland
Source: Stat Med. 2013 Dec 1;33(9):1564–79. doi: 10.1002/sim.6047 (PMC4285225; doi:10.1002/sim.6047)
Supplement: Supplementary file 1 — Supporting info item [file sim0033-1564-sd1.pdf]

Web-based Supporting Materials for

Incomplete contingency tables with censored cells with application to estimating the number of people who inject drugs in Scotland

by Overstall, King, Bird, Hutchinson and Hay

1. Equivalence of posterior inference under Poisson and multinomial models

Forster [1] shows that for complete contingency tables (i.e. when N is known), the joint posterior distribution for $\boldsymbol{\beta}_m$ and m is identical under the Poisson and multinomial formulations when $\pi(\beta_0) \propto 1$, assuming the same prior distribution on the remaining parameters and over the model space. We extend this result here to the case of incomplete contingency tables (i.e. when N is unknown) and in the presence of (uninformative) left censoring. In particular, the posterior distributions are identical under the different model formulations under the prior specifications, $\pi(\beta_0) \propto 1$ and $\pi(N) \propto N^{-1}$ (assuming identical priors on all other parameters and over model space).

First we consider the Poisson formulation given in equation (2.1) of the main manuscript. The full set of model parameters, under model m, is denoted by $\boldsymbol{\theta}_m = \{\boldsymbol{\beta}_m, \sigma^2\}$. We let $\boldsymbol{\phi}_m = \left(\boldsymbol{\alpha}_m, \sigma^2\right)$ (i.e. the set of parameters excluding the intercept term). In addition, for model m (dropping the subscript notation for simplicity) we let $h_i = \eta_i - \beta_0$ (i.e. the linear predictor for cell i, minus the intercept term). From equation (2.5) of the main manuscript, and integrating out the intercept term, the (marginal) posterior distribution of $\boldsymbol{\phi}_m$, m, $\boldsymbol{y}_C$ and $\boldsymbol{y}_U$ is given by

$$\begin{aligned}
\pi(\boldsymbol{\phi}_m, m, \boldsymbol{y}_C, \boldsymbol{y}_U | \boldsymbol{y}_O, \boldsymbol{z}_C) &\propto \pi(\mathbf{z}_C|\mathbf{y}_C)\pi(\boldsymbol{\phi}_m, m) \int_{\mathbb{R}} \pi(\boldsymbol{y}|\boldsymbol{\theta}_m, m)\pi(\beta_0) d\beta_0 \\
&\propto \pi(\mathbf{z}_C|\mathbf{y}_C)\pi(\boldsymbol{\phi}_m, m) \int_{\mathbb{R}} \frac{\prod_{i=1}^{n} \exp(-\mu_i)\mu_i^{y_i}}{\prod_{i=1}^{n} y_i} d\beta_0 \\
&\quad \text{(recalling that } \pi(\beta_0) \propto 1) \\
&= \pi(\mathbf{z}_C|\mathbf{y}_C)\pi(\boldsymbol{\phi}_m, m) \frac{\prod_{i=1}^{n} \exp(h_i)^{y_i}}{\prod_{i=1}^{n} y_i} \\
&\qquad \times \int_{\mathbb{R}} \exp(\beta_0)^N \left(- \exp(\beta_0) \sum_{i=1}^{n} \exp(h_i) \right) d\beta_0.
\end{aligned}$$

Using the substitution $u = \exp(\beta_0)$ and simplifying the expression, we obtain

$$\pi(\boldsymbol{\phi}_m, m, \boldsymbol{y}_C, \boldsymbol{y}_U | \boldsymbol{y}_O, \boldsymbol{z}_C) \propto \pi(\boldsymbol{z}_C|\boldsymbol{y}_C)\pi(\boldsymbol{\phi}_m, m)\frac{\prod_{i=1}^n \exp(h_i)^{y_i}\,(N-1)!}{\prod_{i=1}^n y_i!\,\left(\sum_{i=1}^n \exp(h_i)\right)^N}. \tag{1}$$

Next we consider the alternative multinomial formulation. The posterior distribution of $\boldsymbol{\phi}_m$, m, $\boldsymbol{y}_C$ and $\boldsymbol{y}_U$ is given by,

$$\begin{aligned}
\pi(\boldsymbol{\phi}_m, m, \boldsymbol{y}_C, \boldsymbol{y}_U | \boldsymbol{y}_O, \boldsymbol{z}_C) &\propto \pi(\mathbf{z}_C|\mathbf{y}_C)\pi(\boldsymbol{\phi}_m, m)\pi(\boldsymbol{y}|N, \boldsymbol{\phi}_m, m)\pi(N) \\
&\propto \pi(\mathbf{z}_C|\mathbf{y}_C)\pi(\boldsymbol{\phi}_m, m)\frac{N!}{\prod_{i=1}^n y_i!}\prod_{i=1}^n p_i^{y_i} \times \frac{1}{N},
\end{aligned}$$

substituting the probability mass function for the multinomial distribution. The result immediately follows (i.e. the posterior distribution is identical to equation (1) in this document) by noting that

$$p_i = \frac{\exp(h_i)}{\sum_{j=1}^n \exp(h_j)}.$$

2. Weighted least squares implementation of the Metropolis-Hastings algorithm

In this section we describe the weighted least squares implementation of the Metropolis-Hastings algorithm for GLMs [2] as applied to log-linear models. Let the current parameter values be denoted by $\boldsymbol{\beta}_m$ and define $\mathbf{W}(\boldsymbol{\beta}_m) = \text{diag}\{\mu_i\}$ and $\Delta_i(\boldsymbol{\beta}_m) = (y_i - \mu_i)/\mu_i$, where μ_i is evaluated at the current log-linear parameters, $\boldsymbol{\beta}_m$. Furthermore, we let

$$\begin{aligned}
\tilde{\mathbf{y}}(\boldsymbol{\beta}_m) &= \mathbf{X}_m\boldsymbol{\beta}_m + \boldsymbol{\Delta}(\boldsymbol{\beta}_m); \\
\mathbf{C}(\boldsymbol{\beta}_m) &= \left(\frac{1}{\sigma^2}\boldsymbol{I} + \mathbf{X}_m^T\mathbf{W}(\boldsymbol{\beta}_m)\mathbf{X}_m\right)^{-1}; \text{ and} \\
\mathbf{m}(\boldsymbol{\beta}_m) &= \mathbf{C}(\beta_m)\mathbf{X}_m^T\mathbf{W}(\boldsymbol{\beta}_m)\tilde{\mathbf{y}}(\boldsymbol{\beta}_m).
\end{aligned}$$

The Metropolis-Hastings proposal parameters, $\boldsymbol{\beta}'$ are simulated from,

$$\boldsymbol{\beta}' \sim N\left(\mathbf{m}(\boldsymbol{\beta}_m), \mathbf{C}(\boldsymbol{\beta}_m)\right),$$

and accepted with the standard acceptance probability, $\min(1, A)$, where

$$A = \frac{\pi(\boldsymbol{\beta}'|\boldsymbol{y}, \sigma^2, m)q(\boldsymbol{\beta}_m|\boldsymbol{\beta}')}{\pi(\boldsymbol{\beta}_m|\boldsymbol{y}, \sigma^2, m)q(\boldsymbol{\beta}'|\boldsymbol{\beta}_m)},$$

in which $q(\boldsymbol{\beta}'|\boldsymbol{\beta}_m)$ denotes the multivariate normal proposal density for the proposal values, given the current parameter values (and vice versa).

3. Reversible jump algorithm

Here we consider the reversible jump algorithm [3] to update the model within the MCMC algorithm. We let m denote the current model with associated vector of log-linear parameters $\boldsymbol{\beta}_m$ and design matrix $\boldsymbol{X}_m$. Let $\tilde{m}$ denote the maximal model, i.e. the most complex model we are prepared to consider and $\tilde{\boldsymbol{\beta}}_{\tilde{m}}$ the corresponding posterior mode of the log-linear parameters under the maximal model fitted to the observed cell counts, $\boldsymbol{y}_O$. Note that, for the examples we consider, the maximal model corresponds to the model with all main effects and two-way interactions present. We set $\tilde{\boldsymbol{\eta}} = \boldsymbol{X}_{\tilde{m}}\tilde{\boldsymbol{\beta}}_{\tilde{m}}$ and define the $(n \times n)$ matrix $\tilde{\mathbf{W}} = \text{diag}\left\{\exp(\tilde{\boldsymbol{\eta}})\right\}$.

We propose to move to a model that differs with respect to the current model m by only a single interaction and choose each of these models with equal probability. Suppose that we propose to move to model k, which involves adding an interaction term (i.e. a "birth" move). We let the associated design matrix for model k be $\boldsymbol{X}_k$. We can write $\boldsymbol{X}_k = (\boldsymbol{X}_m, \boldsymbol{S})$ where $\boldsymbol{S}$ is the column vector of the design matrix corresponding to the interaction term that is added to the current model (note that for such moves we also re-order the $\boldsymbol{\beta}$ terms accordingly).

We define,

$$\begin{aligned}
\boldsymbol{P}_k &= \boldsymbol{X}_k\left(\boldsymbol{X}_k^T\tilde{\boldsymbol{W}}\boldsymbol{X}_k\right)^{-1}\boldsymbol{X}_k^T\tilde{\boldsymbol{W}}; \\
C_k &= \left(\boldsymbol{S}^T\tilde{\boldsymbol{W}}\left(\boldsymbol{I} - \boldsymbol{P}_k\right)\boldsymbol{S}\right)^{-1}; \text{ and} \\
m_k &= \boldsymbol{C}_k\boldsymbol{S}^T\tilde{\boldsymbol{W}}\left(\boldsymbol{I} - \boldsymbol{P}_k\right)\tilde{\boldsymbol{\eta}}.
\end{aligned}$$

We simulate $u \sim N\left(m_k, C_k\right)$ and set the proposed model parameters $\boldsymbol{\beta}'_k$ such that,

$$\boldsymbol{\beta}'_k = \begin{pmatrix} \beta'_{(1)} \\ \beta'_{(2)} \end{pmatrix} = \begin{pmatrix} \boldsymbol{I} & -\left(\boldsymbol{X}_k^T\tilde{\boldsymbol{W}}\boldsymbol{X}_k\right)^{-1}\boldsymbol{X}_k^T\tilde{\boldsymbol{W}}\boldsymbol{S} \\ \mathbf{0} & 1 \end{pmatrix}\begin{pmatrix} \boldsymbol{\beta}_m \\ u \end{pmatrix}.$$

Note that $\boldsymbol{\beta}'_{(1)}$ denotes the proposed parameter values for the log-linear terms present in model m and $\beta'_{(2)}$ the parameter value for the interaction term that is proposed to be added.

The move is accepted with probability, $\min(1, A)$, where,

$$A = \frac{\pi(\boldsymbol{\beta}'_k, k|\boldsymbol{y}, \boldsymbol{z}_C, \sigma^2)}{\pi(\boldsymbol{\beta}_m, m|\boldsymbol{y}, \boldsymbol{z}_C, \sigma^2)q(u)},$$

such that q denotes the proposal normal density function with mean m_k and variance C_k. The Jacobian term is simply equal to one and the probabilities of moving between models m and k cancel in the probability so that these terms are omitted in the acceptance probability.

We now consider the case where we move from model k with parameters $\boldsymbol{\beta}'_k$ to model m with parameters $\boldsymbol{\beta}_m$ which involves removing a single interaction term from the model (i.e. a "death" move). The corresponding log-linear parameters in the proposed model are deterministically given by,

$$\boldsymbol{\beta}_m = \boldsymbol{\beta}'_{(1)} + \left(\boldsymbol{X}_k^T \tilde{\boldsymbol{W}} \boldsymbol{X}_k\right)^{-1} \boldsymbol{X}_k^T \tilde{\boldsymbol{W}} \boldsymbol{S} \beta'_{(2)},$$

and $u = \beta'_{(2)}$. Recall that $\boldsymbol{\beta}'_{(1)}$ is the vector of current elements of $\boldsymbol{\beta}'_k$ corresponding to the log-linear parameters present in model m and $\beta'_{(2)}$ is the current value of the log-linear parameter that is removed from the model. This move is accepted with probability $\min(1, A^{-1})$, where A is given above. Finally, note that in both types of model moves (adding or removing an interaction parameter), the hyperparameter σ^2 is not updated within the model move.

4. Additional output

Web Table 1 shows the posterior means of the interaction terms for each year and for the INC-C, REM-C and IGN-C methods. This table acts as a complement to Table 5 (showing posterior probabilities) in the main manuscript.

Web Table 2 shows the posterior mean and 95% HPDIs for the total population size for each year under four different specifications of the prior hyperparameters, a and b, under the proposed INC-C method. The values in this table should be compared against the corresponding values in the first two columns of Table 4 in the main manuscript, where the prior hyperparameters are $a = 0.001$ and $b = 0.001$.

Web Table 1: The marginal posterior means for each two-way log-linear interaction term for the INC-C, REM-C and IGN-C methods. The data-sources are labelled as S1 - social enquiry reports; S2 - hospital records; S3 - Scottish Drug Misuse Database (SDMD) and S4 - HCV diagnosis data-source. An NA indicates that this interaction cannot be identified with the REM-C method.

	2003			2006			2009		
Interaction	INC-C	REM-C	IGN-C	INC-C	REM-C	IGN-C	INC-C	REM-C	IGN-C
S1 × S2	0.00	0.00	0.13	-0.01	-0.00	0.00	0.00	0.01	0.19
S1 × S3	-0.08	-0.09	0.08	0.12	0.14	0.19	0.07	0.09	0.26
S1 × S4	-0.00	NA	-0.01	0.01	NA	-0.00	0.04	NA	0.01
S2 × S3	0.02	0.02	0.19	-0.01	0.00	0.06	-0.04	-0.02	0.17
S2 × S4	0.31	NA	0.27	0.27	NA	0.21	0.18	NA	0.07
S3 × S4	0.01	NA	-0.01	0.01	NA	-0.00	0.01	NA	-0.01
S1 × Age	0.21	0.21	0.25	-0.17	-0.16	-0.21	0.05	0.05	0.19
S2 × Age	-0.04	-0.04	-0.00	0.13	0.13	0.08	-0.24	-0.24	-0.11
S3 × Age	0.09	0.09	0.15	-0.13	-0.12	-0.18	0.01	0.01	0.16
S4 × Age	-0.00	NA	-0.01	0.00	NA	0.01	0.03	NA	-0.01
S1 × Sex	0.09	0.09	0.09	-0.00	-0.00	-0.01	0.00	0.00	0.01
S2 × Sex	-0.00	-0.00	-0.00	0.12	0.12	0.10	-0.13	-0.13	-0.12
S3 × Sex	0.00	0.00	0.00	0.01	0.01	0.00	-0.00	-0.00	0.00
S4 × Sex	-0.01	NA	-0.00	0.00	NA	0.01	-0.00	NA	-0.00
S1 × Region	0.06	0.06	0.07	0.01	0.01	0.04	0.00	0.00	0.01
S2 × Region	-0.16	-0.17	-0.15	0.00	0.00	0.03	-0.00	-0.00	0.01
S3 × Region	-0.00	-0.00	0.00	0.21	0.21	0.24	0.12	0.12	0.14
S4 × Region	-0.14	NA	-0.19	-0.00	NA	-0.10	-0.01	NA	-0.25
Age × Sex	-0.12	-0.12	-0.12	-0.15	-0.15	-0.14	-0.16	-0.16	-0.14
Age × Region	0.19	0.19	0.18	-0.14	-0.14	-0.13	0.13	0.13	0.14
Sex × Region	-0.00	-0.00	-0.00	0.00	0.00	0.00	-0.00	-0.00	-0.00

Web Table 2: Posterior mean (95% HPDI) for the total population size under the INC-C method for each year, for different values of the prior hyperparameters, a and b. The analysis presented in the main manuscript corresponds to $a = b = 0.001$ and the posterior mean (95% HPDI) for the total population size under this analysis (from Table 4) is also shown here for comparison.

Year	$a = 0.001$ $b = 0.004$	$a = 0.001$ $b = 0.002$	$a = 0.001$ $b = 0.001$	$a = 0.001$ $b = 0.0005$	Gelman prior
2003	16300 (14200, 20500)	16500 (14300, 20800)	16700 (14300, 20900)	16700 (14300, 20900)	16500 (14300, 20700)
2006	22800 (15700, 26600)	23200 (19800, 27000)	22900 (16300, 27000)	23000 (19300, 27600)	22900 (18700, 27800)
2009	14600 (11400, 18300)	14600 (11500, 18300)	15600 (11500, 18600)	15200 (11700, 18700)	14600 (11500, 18400)

References

[1] Forster, J. J. (2010). Bayesian inference for Poisson and multinomial log-linear models. *Statistical Methodology*, **7**, 210–224.

[2] Gamerman, D. (1997). Sampling from the posterior distribution in generalised linear mixed models. *Statistics and Computing*, **7**, 57–68.

[3] Forster, J. J., Gill, R. C. & Overstall, A. M. (2012). Reversible jump methods for generalised linear models and generalised linear mixed models. *Statistics and Computing*, **22**, 107–120.
